# Estimating daily semantic segmentation maps of classified ocean eddies using sea level anomaly data from along-track altimetry

**DOI:** 10.3389/frai.2024.1298283

**Published:** 2024-02-22

**Authors:** Eike Bolmer, Adili Abulaitijiang, Jürgen Kusche, Ribana Roscher

**Affiliations:** ^1^Remote Sensing Group, Institute of Geodesy and Geoinformation (IGG), University of Bonn, Bonn, Germany; ^2^Astronomical, Physical and Mathematical Geodesy (APMG) Group, Institute of Geodesy and Geoinformation (IGG), University of Bonn, Bonn, Germany; ^3^Forschungszentrum Jülich, Institute for Bio- and Geosciences Plant Sciences (IBG-2), Jülich, Germany

**Keywords:** sea level anomaly (SLA), deep learning, mesoscale eddies, transformer, radar altimetry data processing, convolutional nerual networks

## Abstract

Mesoscale eddies, which are fast-moving rotating water bodies in the ocean with horizontal scales ranging from 10 km to 100 km and above, are considered to be the weather of the oceans. They are of interest to marine biologists, oceanographers, and geodesists for their impact on water mass, heat, and nutrient transport. Typically, gridded sea level anomaly maps processed from multiple radar altimetry missions are used to detect eddies. However, multi-mission sea level anomaly maps obtained by the operational processors have a lower effective spatiotemporal resolution than their grid spacing and temporal resolution, leading to inaccurate eddy detection. In this study, we investigate the use of higher-resolution along-track sea level anomaly data to infer daily two-dimensional segmentation maps of cyclonic, anticyclonic, or non-eddy areas with greater accuracy than using processed sea level anomaly grid map products. To tackle this challenge, we propose a deep neural network that uses spatiotemporal contextual information within the modality of along-track data. This network is capable of producing a two-dimensional segmentation map from data with varying sparsity. We have developed an architecture called Teddy, which uses a Transformer module to encode and process spatiotemporal information, and a sparsity invariant CNN to infer a two-dimensional segmentation map of classified eddies from the ground tracks of varying sparsity on the considered region. Our results show that Teddy creates two-dimensional maps of classified eddies from along-track data with higher accuracy and timeliness when compared to commonly used methods that work with less accurate preprocessed sea level anomaly grid maps. We train and test our method with a carefully curated and independent dataset, which can be made available upon request.

## 1 Introduction

Detecting ocean eddies is an important task because they can have significant impacts on ocean circulation and marine life. These gyrating currents cause a poleward heat transport and an upwelling of salt and nutrients (Conway et al., 2018) throughout the ocean, influencing the distribution of organisms and the productivity of marine ecosystems. Eddies also amplify sea-air fluxes of water vapor and energy. Therefore, detecting mesoscale eddies can also provide valuable information for weather forecasting and ocean navigation.

Eddies as part of the dynamic ocean can be easily seen and tracked by infrared and other sensors from space. In radar altimetry, sea surface heights are measured that contain features (highs or lows) of an eddy within the satellite footprint. The current research on eddy detection infers results either from gridded sea level anomaly (SLA) maps that are processed from multiple radar altimetry missions (Lguensat et al., 2018), from sea surface temperature (SST) grid maps (Moschos et al., 2020), from both (Zhao et al., 2023), or other grid map observations (Xia et al., 2022). Along-track (AT) altimetry data capture the instant sea-level heights at the time of measurement. In particular, high-frequency ocean variability is well preserved on the AT data. The operational processors generate multi-mission SLA grid maps with a lower effective spatial and temporal resolution compared to their grid spacing and temporal resolution. Thus, compared to smoothed and resolution-degraded two-dimensional altimetry maps, AT data have both spatial and temporal advantages in detecting eddies. SST map products usually have higher resolution but are more unreliable in inferring features that expose eddies, resulting in error-prone eddy detection. Especially for detecting cyclonic eddies of the northern hemisphere from SST maps, there is a lower performance due to weaker cyclonic signatures (Moschos et al., 2020). Conversely, AT data consist of one-dimensional SLA data along with exact spatiotemporal information of each measurement. Although data from multiple ATs could be represented as a two-dimensional grid map, the resulting problems such as cross-track errors and areas without SLA information and, therefore, sparse feature maps impede the processing within conventional neural networks. For example, these problems would lead to high gradients within a two-dimensional grid map in conventional convolutional neural networks (CNNs) such as a U-Net as they rely on data without gaps or jumps. Moreover, it poses a challenge to use the spatiotemporal information of each sample in the map.

**Figure 1:**
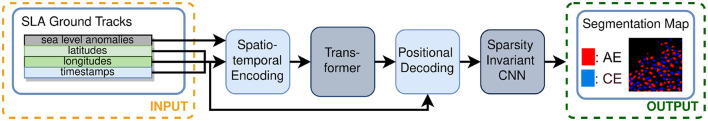
Overview of Teddy. The along-track data with its spatiotemporal information are the input that are fed first into a Transformer module to process the spatiotemporal context (see Section 4.1). The resulting sparse feature map that is inferred in the positional decoding (see Section 4.2) is then processed in a sparsity invariant convolutional neural network (see Section 4.3) to finally generate a segmentation map of classified eddies.

In this study, we aim to answer the research question of whether it is possible to utilize raw observational AT data instead of relying on highly correlated and preprocessed two-dimensional SLA grid maps for the accurate detection and classification of mesoscale ocean eddies. For this study, we infer a two-dimensional segmentation map of classified cyclonic (*CE*) and anticyclonic eddies (*AE*) from raw observational AT SLA data. We aim to tackle the aforementioned challenges that come with AT data, such as cross-track errors and varying sparsity, by avoiding the use of conventional CNNs. Instead, we use a Transformer module for encoding the spatiotemporal information of the tracks and a sparsity invariant CNN. This model is trained on a dataset that is first generated by the *py-eddy-tracker* algorithm and then carefully curated by photointerpretation of SLA and SST grid maps for the removal of falsely annotated eddies. We support our claims by testing our method's results, which include not only segmentation maps but also semantics, only on AT positions. This evaluation is carried out using metrics such as the Dice score, recall, and precision on independent reference data. It is generated through photo interpretation of a time series of SST grid maps overlaid on eddy candidates produced by a preliminary CNN that uses SST data as an input. See Figure 1 for an overview of Teddy's architecture.

To summarize, our approach is able to (i) show that cleaning the error-prone reference data using photointerpretation of SLA and SST grid maps leads to improved model training and eddy classification from SLA ground tracks, (ii) infer daily two-dimensional segmentation maps of classified eddies from one-dimensional AT data in near real-time with higher accuracy than using conventional methods by exploiting the spatiotemporal context from coordinates and timestamps of the observations, and (iii) deal with ground tracks of different lengths. These claims are supported in this study by our experimental evaluation in Section 5.

While powerful methods, such as the U-Net architecture (Ronneberger et al., 2015) or the Vision Transformer (Dosovitskiy et al., 2020), exist for semantic segmentation of images or other two-dimensional grid maps in deep learning, they rely on processed data where information about fine structures, such as eddies, dissipates. To our knowledge, this study represents the first instance of directly employing AT radar altimetry data for eddy detection prior to gridding. This novel approach we propose in this study therefore significantly enhances the field of eddy detection and classification.

## 2 Related work

### 2.1 Eddy detection and classifying

Automatic eddy detection can be traced back to the development of oceanographic research and the use of various remote sensing techniques. Traditional methods mainly use algorithms that are either geometrical contour-based or physical parameter-based. A popular representative of physical parameter-based algorithms is the Okubo-Weiss parameter method (Weiss, 1991), which demands high expert knowledge and region-specific parameters to ensure accurate detection. In addition, the results are sensitive to noisy sea-level anomaly data (Chelton et al., 2007). In terms of geometric contour-based methods, Chelton et al. (2011) conducted the most notable research. Closely related to it is the *py-eddy-tracker* (Mason et al., 2014) that was used to generate the reference data used in this study.

A recent approach for our analysis involves the use of CNNs, which have already been used to detect eddies using two-dimensional SLA or sea surface height (SSH) (Lguensat et al., 2018; Santana et al., 2020), sea surface temperature (SST) (Moschos et al., 2020, 2022b), both (Zhao et al., 2023), or other grid map observations (Xia et al., 2022). In most cases, such as in the study of Lguensat et al. (2018), a special CNN, known as U-Net, has been used, which is a state-of-the-art method for semantic segmentation of two-dimensional data has been used. A similar U-Net was presented in a study conducted by Franz et al. (2018) among others comparing itself to another neural network architecture.

However, these methods rely on two-dimensional SLA grid maps as input data, which is problematic since operational processors create multi-mission (processing level 4) SLA grid maps with an effective spatiotemporal resolution far lower than their grid spacing and temporal resolution. The creation of SLA grid maps can lead to error-prone eddy detection, as mentioned in the study of Lguensat et al. (2018). Another possibility for eddy detection is the usage of SST maps. Although SST grid maps are generally available in higher resolution, the products can have area-wide gaps due to clouds or may represent an analysis of the daily average SST from multiple sources (CMEMS, 2021). Comparing SLA and SST data in the same area shows some degree of discrepancy between eddy appearances and positions. Studies, such as Moschos et al. (2022b), that rely on simulated data where modalities such as SST and SLA are simulated with the same high accuracies show that groups of eddies can hide behind one single falsely detected eddy in the SLA data of much lower effective resolution. Owing to the disagreement in the data, different and independent reference data of eddies should be used for eddy detection.

### 2.2 Eddy reference data generation

There is a scarcity of independent and accurate reference data, mainly because physical parameter-based or geometry-based algorithms from independent data sources, such as from SST grid maps, can generate eddy center positions (Dong et al., 2011); however, for reference data generation, eddy shapes are also required.

Architectures, such as the *EddyNet*, from other studies (Lguensat et al., 2018) are trained and evaluated with annotated data that are generated using conventional eddy detection algorithms such as the *py-eddy-tracker* that relies on the Okubo-Weiss parameter method (Weiss, 1991) on height maps such as SSH or SLA grid maps. While the method is well established and utilized in ocean data products (Pegliasco et al., 2022), the effective resolution of the input data that the method relies on is between 150 km to 200 km in the gulf stream area [see the quality information document of CMEMS (2023)]. This spatial resolution is far lower than their grid spacing of 1° and less than even larger eddies. Furthermore, due to the accumulation of data over multiple days, the effective temporal resolution is lower than the provided temporal product resolution of 1 day. When used in any eddy detection method, an unknown number of eddies will go undetected, while an unknown number of so-called ghost eddies will be detected. These ghost eddies occur when smaller structures aggregate into one smooth object that can be easily mistaken for an eddy.

### 2.3 Transformer and signal processing

The effective resolution of SLA AT data is higher than the one from the SLA grid maps, but, due to its different modality, the intrinsic spatial context of a regular grid map is missing and therefore cannot be used in eddy detection methods that utilize them in CNNs, as demonstrated by Lguensat et al. (2018). To utilize the spatiotemporal context that is occurring in AT data, we use the Transformer module instead.

Transformers come from the field of Natural Language Processing (NLP) (Vaswani et al., 2017), which suit the modality of one-dimensional AT data since both process arrays of data of varying intrinsic contexts to each other. In the field of image-based analysis and interpretation, this methodology was first introduced with the Vision Transformer by Dosovitskiy et al. (2020) and demonstrated the abilities of Transformers by outperforming a state-of-the-art neural network on numerous classification tasks within the field of remote sensing (Aleissaee et al., 2023) and environmental sciences (Mousavi et al., 2020; Yang et al., 2022). The latter utilizes the SWIN Transformer (Liu et al., 2021) that became popular for combining the benefits of both Vision Transformers and CNNs in image recognition tasks.

In the field of deep learning, one particularly notable method for one-dimensional signal processing is the long short-term memory (LSTM) network (Hochreiter and Schmidhuber, 1997). This network has demonstrated its ability to achieve state-of-the-art results using unprocessed one-dimensional data (Rußwurm and Körner, 2020).

### 2.4 Sparsity invariant CNNs

The Transformer encoder output has the same dimensionality as the input, which is a one-dimensional array of AT SLA data, expanded to a second dimension corresponding to the dimensionality of the Transformer module. For every data point in this array, latitudes, longitudes, and timestamps are also available and are used to transform the AT SLA data into a grid with Cartesian coordinates as a preparation for following methods that lead to a desired two-dimensional segmentation map. Studies such as Liu et al. (2018) demonstrate that CNNs are unable to model the coordinate transform task. They offer a solution by concatenating coordinate channels to the input layer, but this approach is insufficient for our sparse data in this study.

Additionally, due to the sparsity of AT data within our study site, a resulting two-dimensional feature map will also be sparse. Even though a conventional two-dimensional convolutional neural network will struggle with this map, studies such as that of Zweig and Wolf (2017) introduce the ability to interpolate data gaps within the two-dimensional array. Jampani et al. (2016) used bilateral filters to handle inputs of irregular sparsity, but this approach requires guidance information and is computationally expensive. Uhrig et al. (2017) introduced weighting to the convolutional layer depending on the sparsity itself. This weighting results in an invariancy to the sparsity of the data. Since our AT data can vary a lot in the sparsity, it is deemed to be useful and will be investigated in our experiments.

## 3 Data

### 3.1 Study site

#### 3.1.1 Sea level anomaly along-track data

For this study, AT data of SLA from the Copernicus Marine Environment Monitoring Service (CMEMS, 2020) were used from 00:00:00 h of 1 January 2017 to 23:59:00 h of 31 December 2019. This data product processes data from all altimeter missions available to CMEMS, including various Sentinel and Jason missions as well as the Saral/AltiKa, Cryosat-2, Topex/Poseidon, ERS-1, ERS-2, and Envisat missions. The chosen region is within the western Gulf stream area of the northern Atlantic with latitudes Φ^min^ = 16°N and Φ^max^ = 56°N and longitudes Λ^min^ = 260° and Λ^max^ = 320°. A sample of these data is shown in Figure 1 in which 1 test day (7 January 2017) is shown with all AT data within a time frame of ± 7 days that is used as an input into our architecture. The ground tracks of the AT data depend on the different satellite orbits, which result in varying coverage and sparsity of the data in every considered area and day. Within one AT, two observations are typically distanced from each other by approximately 0.05° or 5km on the ground track with approximately 10s of time difference. In this region, there is a large occurrence of dynamic mesoscale eddies (Dong et al., 2022) along the gulf stream as well as in different water bodies such as the Gulf of Mexico.

#### 3.1.2 Sea level anomaly and sea surface temperature grid map data

For the original reference data generation, we used SLA grid maps. These are daily gridded (Level 4) sea level anomaly data from multiple missions from CMEMS with a spatial resolution of 0.25° × 0.25° from the same time and region (CMEMS, 2023).

For our photointerpretation methods, we used SST data from CMEMS (2021) within the same study area. With 0.05° × 0.05°, these grid maps have a much higher resolution, and they will aid as additional information in the photointerpretation steps described in Sections 3.2.1 and 3.2.2.

### 3.2 Annotation generation

For model training and evaluation, we created two datasets. The first reference dataset is created based on SLA data and the *py-eddy-tracker*. It is annotated with the three classes [*0: No Eddy (NE), 1: Anticyclonic Eddy (AE), 2: Cyclonic Eddy (CE)*]. We further reduced the number of false positives of the AE and CE classes within this *py-eddy-tracker* product, which includes ghost eddies. This method is described in Section 3.2.1. To further evaluate our method, we created an independent reference dataset that is not based on the *py-eddy-tracker* and an SLA grid map product. For this dataset creation, we used SST data and photointerpretation, as described in Section 3.2.2.

#### 3.2.1 Training and validation annotation data generation

**Figure 2:**
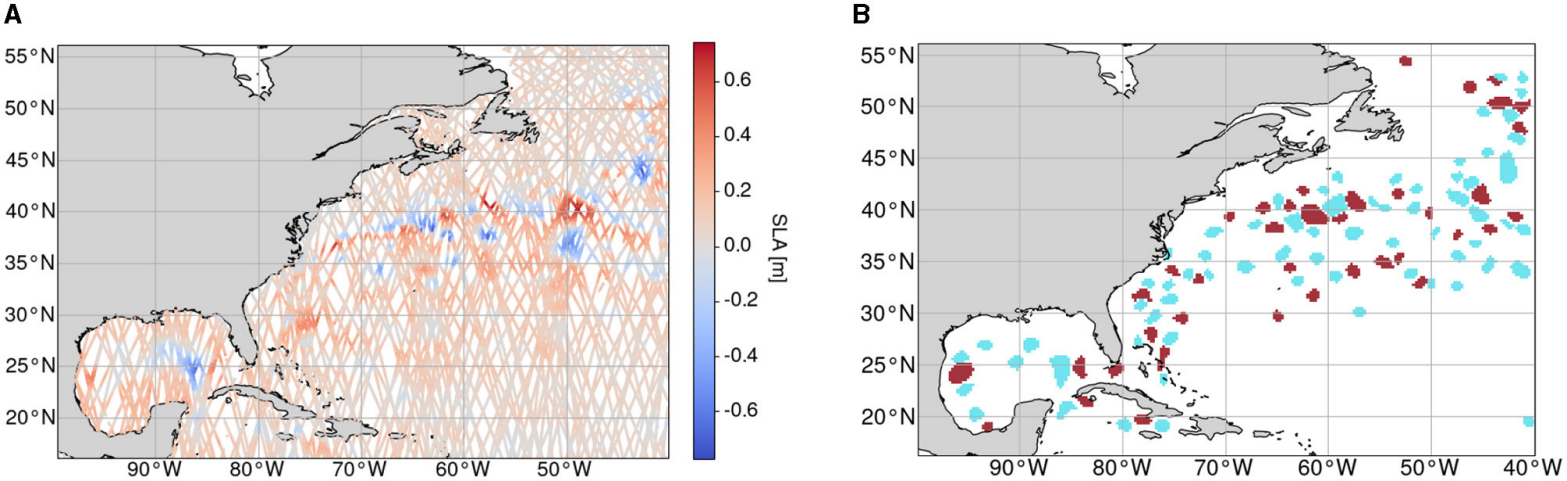
Sample of data from 7 January 2017 12:00 ± 7 days within the study area with the borders Φ^min^ = 16°N, Φ^max^ = 56°N, Λ^min^ = 260° and Λ^max^ = 320° from which input data are sampled along with the respective reference data. For this time frame, 83,000 observations are available. **(A)** Along-track sea level anomaly data of the whole study area within a time frame of 7 January 2017 ± 7 days. **(B)** Reference data produced by utilizing the *py-eddy-tracker*. Blue: Anticyclonic Eddy (*AE*), Red: Cyclonic Eddy (*CE*).

In addition to the SLA data, as described in Section 3.1.2, we created a corresponding set of segmentation masks for training and validating our framework. For that, we use the *py-eddy-tracker* on SLA data which is a geometric-based method to infer the boundaries of the eddies. The contours are transformed into indexed segmentation maps with three classes: 0: *No Eddy (NE)*, 1: *Anticyclonic Eddy (AE)*, and 2: *Cyclonic Eddy (CE)*. These annotations are available in the form of a two-dimensional segmentation map that we utilize completely. As mentioned earlier, we train and validate our architecture on reproduced and predicted two-dimensional shapes of eddies. Hence, we not only train and use annotations on the ground tracks of the AT data alone but also train a model for a segmentation grid map that can be valuable for future analysis. A sample day of reference data throughout the whole study area can be seen in Figure 2.

**Figure 3:**
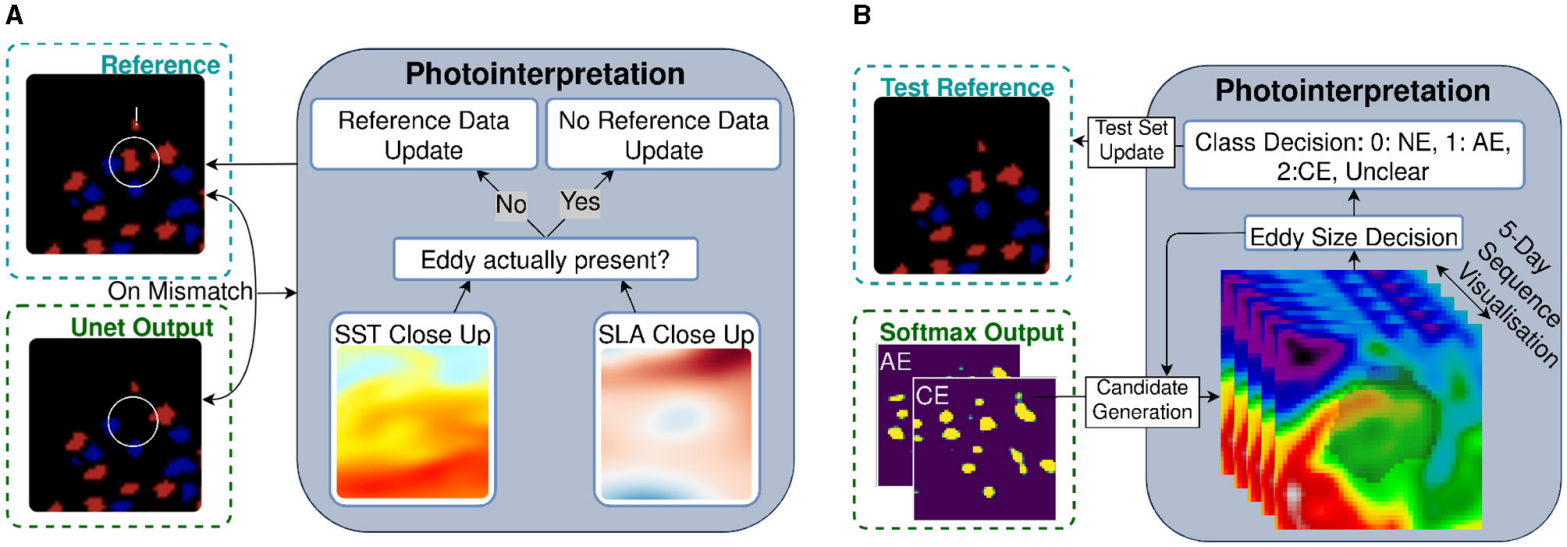
Overviews of the reference data cleaning process and test reference generation process both using photointerpretation. **(A)** Reference data cleaning process using photointerpretation. A U-Net model output trained on our basis reference dataset is matched to the same reference data. If there is a mismatch, manual photointerpretation will be performed with the help of SST and SLA data to decide on the removal of the eddy. **(B)** Generation process of the reference data for testing. Neural network models create candidates of eddies of different classes. With manual photointerpretation, the size of the eddy can be adjusted and a label can be assigned. Afterwards, the results will be added to the test reference dataset.

This reference dataset is used for training and validation purposes only. As mentioned earlier, these data are produced from two-dimensional SLA grid maps, which can lead to errors in eddy detection. We reduced the errors by a process using a photointerpretation method (see Figure 3 for an overview).

The first predictions of segmentation maps are produced by a U-Net CNN (Bolmer et al., 2022) similar to that used by Lguensat et al. (2018), trained on the data from this study area. Then, every eddy in the reference data is compared to the prediction. If no pixel of one class in the model output is present in the prediction, this mismatch will be evaluated manually to determine whether there is indeed an eddy at this location. A close-up of the supposed eddy is shown with its SST and SLA data to aid the decision. If the photointerpretation finds no eddy, the reference data are updated.

With this method, we cleaned the reference data from false negatives of class *NE* as well as false positives of classes *AE* and *CE*, which corresponds to the mentioned ghost eddies. In total, we removed 2.1% of *AE* and 1.3% of *CE* annotations. However, this reference dataset still originates from and depends on the *py-eddy-tracker* output. This introduces bias, rendering the dataset suboptimal as a test dataset to fairly compare methods with each other. For a smaller but independent reference dataset, we use a different method, as described in the following Section 3.2.2.

#### 3.2.2 Test data generation

For testing our approach and comparing it to other methods, an accurate and independent test dataset is produced. This product consists of daily two-dimensional segmentation maps of the three classes 0: *No Eddy (NE)*, 1: *Anticyclonic Eddy (AE)*, and 2: *Cyclonic Eddy (CE)* within the testing region from 1 January 2017 to 4 February 2017. In this method, we use photointerpretation on eddy candidates produced by a U-Net that utilizes the SST grid-map data as an input in addition to the output generated by AT SLA data. An overview of the test data generation method is shown in Figure 3.

For both eddy classes, we first generate two grid maps each from an additional trained U-Net model output that uses SST grid maps as an input. These grid maps are produced with a *softmax* layer at the end in a way that they represent a probability of each pixel or grid point for the classes *AE* and *CE*. With a given probability threshold, candidates within the region are shown individually on top of the currently considered day of SST data for photointerpretation. The threshold affects the eddy area since the predictions of the grid point's probabilities are either included or excluded to the candidate. To this extent, the probability threshold is adjustable to be able to align the prediction with the visually assessed eddy size. For easier interpretation, the past and future 2 days of SST grid maps can be shown as well. With this visualization, it can be decided if the given candidate's pixels should be annotated as *NE, AE, CE*, or *Unclear*. After displaying all candidates, a blank test reference dataset is updated, and the next day's iteration can be presented to the interpreter.

We decided to annotate eddies only when there is no uncertainty for the interpreter, which will lead to a number of missing eddies but makes sure that there are no false positives. As a result, a model will tend to predict a larger number of eddies than what is present in the reference data. This will lower the Dice score, making it less informative compared to a metric such as recall. The recall metric is not influenced by false positives but rather focuses on the true positives of a class and their alignment with the prediction. We therefore will evaluate all mentioned metrics and expect a segmentation performance that is represented by Dice score with even larger values for the recall, since it benefits from the method's ability to predict the manually detected existing eddies.

## 4 Model architecture

To infer two-dimensional segmentation maps of classified eddies from ATs, we introduce several modules (see Figure 1 for an overview of all modules). We further define a 4 × *G*-dimensional matrix *X*^(0)^ = [^**x**^(0)^, **x**^t^, **x**^lat^, **x**^lon^]*T*^ that represents each AT and consists of SLA data **x**^(0)^ each with a time **x**^t^, latitude **x**^lat^, and longitude stamp **x**^lon^.

First, the spatiotemporal context of the AT data is encoded and processed with a Transformer module (Section 4.1). The encoded features *X*^(3)^ are transformed into a feature map *X*^(4)^. With its first two dimensions, this map corresponds to the desired two-dimensional segmentation map covering the currently considered two-dimensional sea surface area using the latitude and longitude information from the observation data. An additional third dimension is used for the features from the Transformer module to be stored for each grid position.

This feature map *X*^(4)^ is then fed into a sparsity invariant CNN module (Section 4.3) that decodes the feature map into a two-dimensional segmentation map of classified eddies. Utilizing a sparsity invariant CNN is necessary due to the resulting sparsity of the map since not every grid point will have been covered by at least one ground track observation, and the sparsity itself can vary a lot, depending on how many satellites were observing within the given time frame and region.

### 4.1 Transformer module

**Figure 4:**
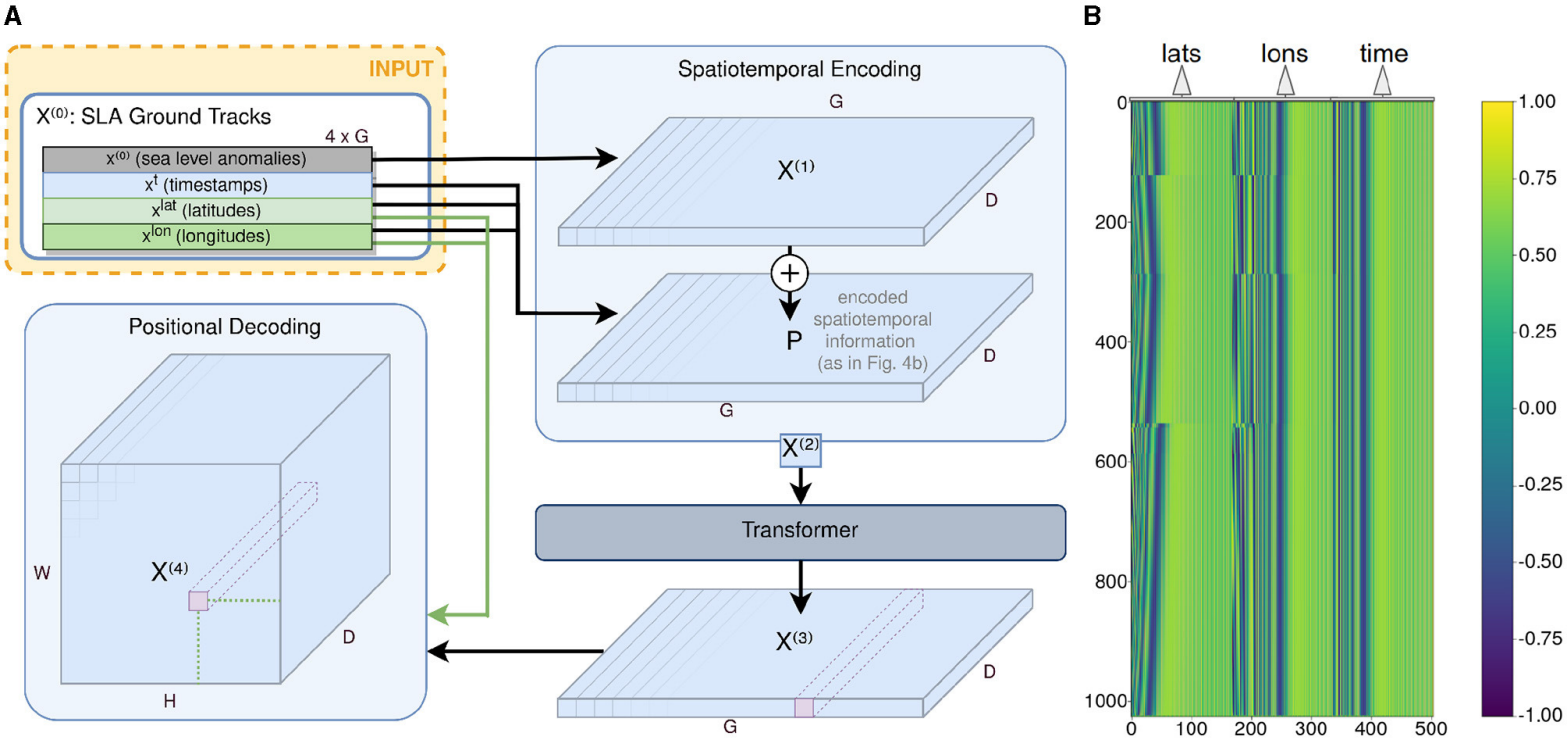
Transformer module architecture overview and visualization of the positional encoding. **(A)** Overview of the Transformer module along with the positional encoding before and the positional decoding after the Transformer encoder. The positional decoding transforms the two-dimensional features (*G* × *D*) into a two-dimensional grid map (*H* × *W*) with its features spanning into a third dimension of size *D*. **(B)** Visualization of a sample of the positional encoding matrix *P*. Here: *D* = 512, *G* = 1024. Each row represents the positional encoding of one observation *g* ∈ *G*.

The Transformer module is introduced to process the vector of SLA data **x**^(0)^ ∈ ℝ^1 × *G*^ along with its spatiotemporal information that is a time stamp **x**^t^ ∈ ℝ^1 × *G*^ as well as latitude **x**^lat^ ∈ ℝ^1 × *G*^ and longitude **x**^lon^ ∈ ℝ^1 × *G*^ of the satellite's trace during respective observations *g* ∈ [1…*G*]. For an overview of this module (see Figure 4).

A first linear layer is applied on **x**^(0)^ that expands this one-dimensional input vector of SLA ground track observations with the length *G* to the dimension *G* × *D* of the Transformer model dimension resulting in a matrix *X*^(1)^ ∈ ℝ^*G* × *D*^ as the output.

To provide the Transformer with the information on the time and location of the input data, it is processed in a spatiotemporal encoding similar to the positional encoding introduced by Vaswani et al. (2017). The spatiotemporal information consists of three vectors **x**^lat^, **x**^lon^, and **x**^t^ of length *G*. Each vector is being normalized to values between 0 and 1 according to their given boundaries [ϕ^min^, ϕ^max^] ∈ [Φ^min^…Φ^max^] and [λ^min^, λ^max^] ∈ [Λ^min^…Λ^max^] (3.1.1). From that normalization step, it follows that only relative times and positions are considered. Then, each spatiotemporal coordinate $\phi_{g}\in {\text{x}}^{\text{lat}}$, $\lambda_{g}\in {\text{x}}^{lon}$ and $t_{g}\in {\text{x}}^{t}$ is used to calculate an encoded vector ${\text{p}}_{g}^{\text{pos}}$ of length $\left\lfloor \frac{D}{3}\right\rfloor$ with the sinusoidal functions


(1) $$\begin{array}{l} \begin{array}{c} {\text{p}}_{g,2i}^{\theta }=\sin\left(\frac{\theta }{10000^{\frac{2i}{\left\lfloor \frac{D}{3}\right\rfloor }}}\right)\\ {\text{p}}_{g,2i+1}^{\theta }=\cos\left(\frac{\theta }{10000^{\frac{2i}{\left\lfloor \frac{D}{3}\right\rfloor }}}\right), \end{array} \end{array}$$

with *i* ∈ [1…⌊*D*/3⌋] and θ ∈ [ϕ, λ, *t*]. The resulting vectors ${\text{p}}_{g}^{\phi }$, ${\text{p}}_{g}^{\lambda }$ and ${\text{p}}_{g}^{t}$ are concatenated to a vector **p**_*g*_ of length *D*. If *D*/3 is not an integer, the vector will be padded with zeros to reach the dimension *D*. The padding is done for every observation *g* ∈ [1…*G*], resulting in the positional encoding matrix *P* ∈ ℝ^*G* × *D*^ that represents each spatiotemporal coordinate of the ground track observations (see Figure 4).

In a next step, the positional encoding *P* will be added to the Transformer input with a factor *q*:


(2) $$\begin{array}{l} X^{\left(2\right)}=X^{\left(1\right)}+qP \end{array}$$

The resulting matrix *X*^(2)^ is the input of the Transformer encoder module $T_{L,N,D}$. It consists of *L* identical Transformer layers that each have *N* heads in the multi-head attention block. The number of expected features *D* sets the dimension of the linear layers and the Transformer model:


(3) $$\begin{array}{l} X^{\left(3\right)}=T_{L,N,D}\left(X^{\left(2\right)}\right) \end{array}$$

A layer normalization is applied after each Transformer layer.

### 4.2 Positional Decoding

The output of the Transformer *X*^(3)^ ∈ ℝ^*G* × *D*^ is decoded into *D* grid maps that correspond to the area that is being observed within the given latitude and longitude boundaries ϕ^min^, ϕ^max^, λ^min^ and λ^max^.

For that decoding, a tensor *X*^(4)^ ∈ ℝ^*D* × *H* × *W*^ with grid map height *H* and width *W* is calculated that has the entries


(4) $$\begin{array}{l} {\text{x}}_{h,w}^{\left(4\right)}=\frac{\overset{G}{\underset{g=1}{\sum }}{\text{x}}_{g}^{\left(3\right)}\left(\left\lceil \phi_{g}H\right\rfloor =h,\left\lceil \lambda_{g}W\right\rfloor =w\right)}{O_{h,w}}, \end{array}$$

with ${\text{x}}_{g}^{\left(3\right)}\in X^{\left(3\right)}$ of each observation *g* being summed up if its rounded spatial coordinates ⌈ϕ_*g*_*H*⌋ and ⌈λ_*g*_*W*⌋ match with the grid point coordinates *h* and *w* and being averaged independently by dividing the sum by the number of occurrences *O*_*h, w*_ at this grid point. The Transformer depth *D* is preserved with the expectation that important features are encoded for the respective position on the grid map.

### 4.3 Sparsity invariant CNNs

**Figure 5:**
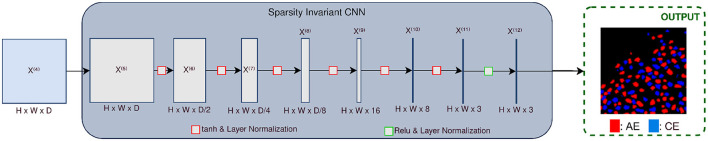
Overview of the sparsity invariant CNN module. The input is a *D* × *H* × *W* sparse feature map which is being decoded into a *H* × *W* segmentation map *X*^(13)^ of classified eddies. Each sparsity invariant convolutional layer reduces the dimension *D* until *D* = 3 dimensions represent the probabilities of the three classes for every grid point *H* × *W*. Between each layer, the *tanh* activation function and layer normalization are applied.

The resulting tensor can vary in its sparsity, depending on the chosen AT data size *G*, how much area is covered by land, and what resolution the AT observations were taken. In this case, a conventional CNN decoder would generate pronounced gradients and high values from features representing gaps. These elevated values could overshadow pertinent features within the network, rendering their extraction more challenging. Therefore, a sparse invariant CNN module (Uhrig et al., 2017) is being introduced (see Figure 5).

The input is a tensor *X*^(5)^ ∈ ℝ^*D* × *H* × *W*^, and in each of the eight layers, the tensor will be reduced in depth until the output *X*^(13)^ ∈ ℝ^3 × *H* × *W*^ can infer a segmentation map with three classes (0: no eddy, 1: *AE*, 2: *CE*).

Each layer considers a sparsity mask and, depending on the occupancy of the mask, the layer will be normalized and the mask will be updated with a max pooling operation in the same way as described in Uhrig et al. (2017). After each layer except the last, the tanh activation function and layer normalization are applied.

## 5 Experiments

For the experiments conducted in this study, the Teddy architecture is fully utilized as described in Section 4 and shown in Figure 1. The architecture contains the Transformer (see Section 4.1) for treating the AT data in a spatiotemporal context and a sparsity invariant CNN module (see Section 4.3) that processes the feature map of varying sparsity into a segmentation map of classified eddies. The setup of Teddy and comparison methods in our experiments are specified in Section 5.1. The results are compared to the methods in other studies described in Section 5.2, and the performance and advantage of using each block are evaluated in Section 5.3.

### 5.1 Experimental setup

#### 5.1.1 Teddy architecture setup

For the choice of parameters for the architecture setup of Teddy and its training procedure, we undertook an empirical investigation involving the systematic manipulation of the hyperparameter values to assess their influence on the model's performance when evaluated on our validation dataset. The training process of the model was carried out by optimizing the Dice-loss using the *AdamW* optimizer with a learning rate of 10^−5^, a batch size of 8, and a dropout rate of 5%. Convergence of both the loss values from the training set and the Dice scores of the validation set was achieved after 3,800 epochs.

For the input of our architecture Teddy, we choose a number of *G* = 4, 000 observations, which is a compromise between two factors. On the one hand, a higher number leads to a larger attention map that represents calculated weights of each observation to each other one within the Transformer $T_{L,N,D}\left(X^{\left(2\right)}\right)$ and therefore grows quadratically in computations with every additional observation. On the other hand, a lower number leads to a sparser feature map due to a lower density of observations that covers the considered region. The area that is considered for the input is limited by *H* = *W* = 64 grid points, each with a resolution of 0.25°.

The positional encoding *P* is added to the input with the factor *q* = 0.1. Own experiments show that a larger factor does not lead to a convergence of the training, assumingly because the positional encoding is shifting the data within the feature space too much to be interpretable by the model. The Transformer itself consists of *D* = 256 dimensions with *L* = 3 individual blocks that each have *N* = 8 parallel multi-head attention layers that suffice the task of processing the SLA observations together with its spatiotemporal information.

The sparsity invariant CNN module consists of eight layers. The input *X*^(5)^ with dimension 256 × 128 × 128 ensures that the features of the Transformer output are preserved. All convolutional layers and the sizes of this setup are shown in Figure 5.

#### 5.1.2 Comparison methods

For comparison, we additionally utilized a number of state-of-the-art methods of different complexity. For two-dimensional semantic segmentation, we conducted our experiments with the commonly used method of the geometry-based *py-eddy-tracker* algorithm as described in Section 3.2.1. Furthermore, we employed a U-Net architecture that resembles the *EddyNet* and is set up and trained as described by Lguensat et al. (2018). Both methods are applied on processed two-dimensional SLA grid map data.

To our knowledge, there are no comparable studies that use one-dimensional AT radar altimetry data directly to infer two-dimensional segmentation maps. However, we also employed methods that infer point-wise segmentation and classification of one-dimensional data. This includes a generic one-dimensional CNN that consists of eight consecutive modules each with a convolutional layer with kernel size 5 and a *ReLU* activation function. It is trained by optimizing the Dice-loss using the *AdamW* optimizer with a learning rate of 10^−3^ and a batch size of 8. Additionally, we trained and tested an LSTM model similar to that of Rußwurm and Körner (2020), which encodes the SLA AT data along with its normalized spatiotemporal information in one setup with four bidirectional and in another setup with four unidirectional LSTM layers. The hidden states are set up to contain 220 features, and their forward and backward passes are concatenated to be fed into one dense linear output layer to segment and classify each AT data point. Furthermore, to compare the one-dimensional segmentation output to a method of low complexity, we implemented a k-nearest neighbors (k-nn) algorithm with Euclidean distances and *k* = 75, which we found best suited by empirical investigation. The input length of the AT data throughout all comparison methods is again *G* = 4, 000.

### 5.2 Result evaluation compared to different methods

**Figure 6:**
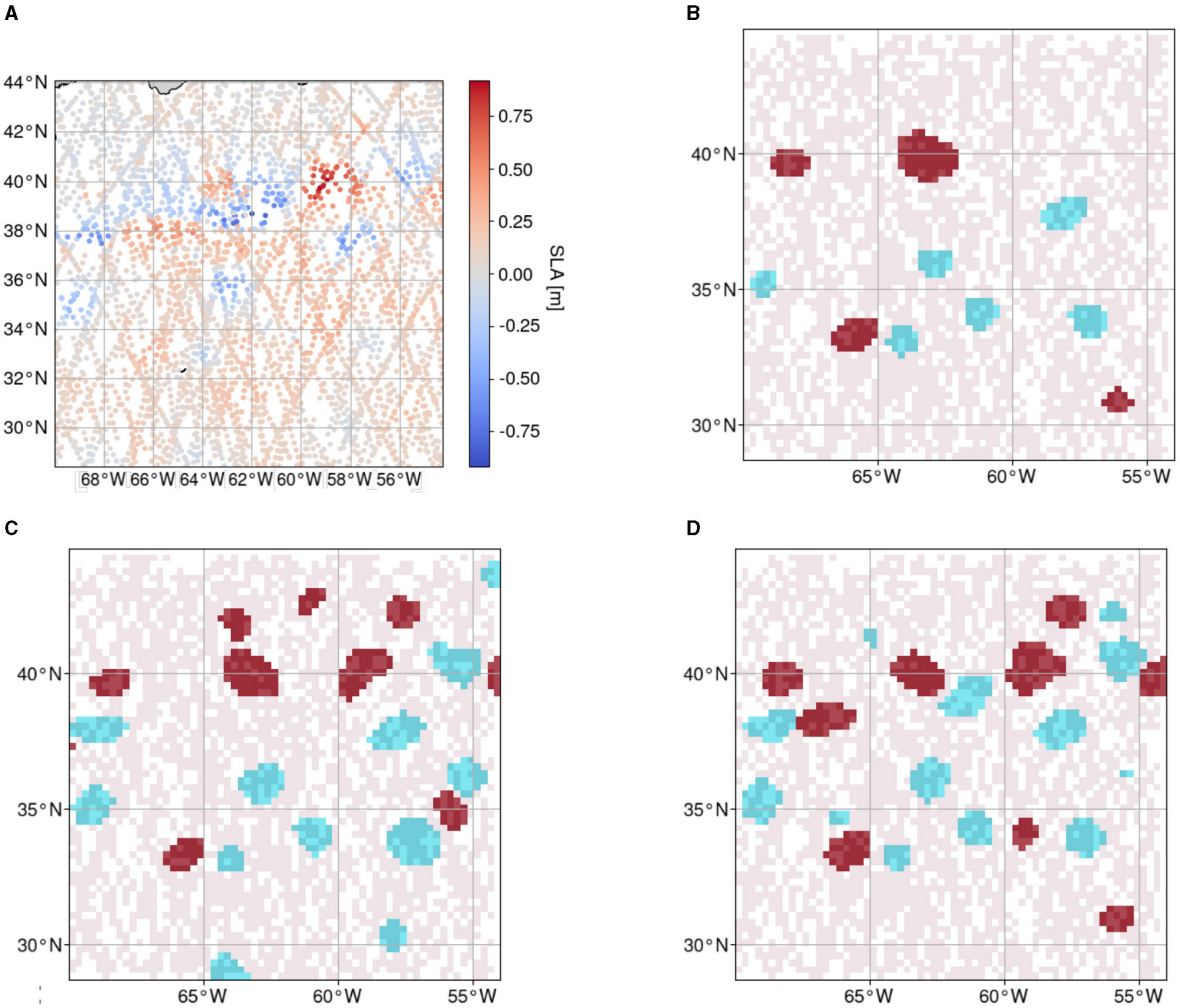
Input observations, sparse feature map, test and training reference data, and prediction of one sample day (10.01.2017) of the region chosen for evaluation. **(A)** Each dot represents one observation of sea level anomaly with the respective timestamp and coordinates within the given spatial and temporal (± seven days) constraints. **(B)** Reference data for testing retrieved by photointerpretation (red: *AE*, blue: *CE*) overlayed on the sparse feature map (light red). **(C)** Reference data for training retrieved with the *py-eddy-tracker* (red: *AE*, blue: *CE*) overlayed on the sparse feature map (light red). **(D)** Prediction of Teddy (red: *AE*, blue: *CE*) overlayed on the sparse feature map (light red).

With this setup, the model is trained on the dataset from 16 March 2017 to 31 December 2019 with annotations generated as described in Section 3.2.1 and is tested on the data from 1 January 2017 to 4 February 2017 generated as described in Section 3.2.2. A sample prediction with the respective input AT observations is shown in Figure 6.

We compared our results with the results from the *py-eddy-tracker*, the *EddyNet* (Lguensat et al., 2018), and a generic CNN. All methods are evaluated on two-dimensional reference data. For methods that use AT data as an input, the semantics only on the tracks are additionally evaluated. As a basis, the class-wise and mean Dice score, precision, and recall are calculated. Optimally, training on the cleaned reference data would lead to a decreased amount of false negatives, i.e., pixels, where the model predicts *NEs* that were labeled as *AEs* or *CEs* in the reference data since for the cleaned reference data set it was attempted to be freed of wrongly labeled *AEs* and *CEs*. The test data are generated with the aim to have a high rate of occurring *AEs* or *CEs* at the cost of some missing eddies that were too uncertain to annotate as such. The recall is sensitive to a change in these false negatives and makes it an important metric to consider. An overview of all resulting metrics with the different methods can be seen in Table 1.

**Table 1:** Performances of different works [Teddy, *py-eddy-tracker* (Mason et al., 2014), and *EddyNet* (Lguensat et al., 2018) for two-dimensional segmentation map outputs as well as an LSTM model (Rußwurm and Körner, 2020), a baseline CNN, and a k-nearest neighbors algorithm for one-dimensional segmentation outputs].

**Method**	**Dice score [*%*]**	**Precision [*%*]**	**Recall [*%*]**
Teddy	59.3 (92.2, 39.0, 46.5)	58.8 (93.4, 42.3, 40.8)	66.9 (91.3, 46.9, 62.6)
Py-Eddy-Tracker	57.1 (91.3, 39.8, 40.1)	56.6 (93.2, 42.7, 33.8)	65.0 (89.7, 47.9, 57.3)
EddyNet	55.8 (91.7, 36.1, 39.8)	56.7 (92.8, 43.6, 33.7)	61.6 (90.7, 38.5, 55.7)
Teddy (AT only)	59.4 (91.8, 39.3, 47.0)	58.8 (93.1, 42.6, 40.8)	67.2 (90.8, 47.3, 63.4)
LSTM (AT only)	49.0 (86.3, 25.7, 35.0)	47.6 (91.0, 25.6, 26.1)	57.6 (82.2, 30.0, 60.6)
1D CNN (AT only)	36.8 (80.3, 16.7, 13.3)	38.2 (90.4, 15.2, 9.1)	41.5 (72.5, 23.8, 28.0)
k-nn (AT only)	46.5 (92.9, 9.8, 36.7)	54.8 (89.4, 32.0, 43.1)	44.8 (96.7, 5.8, 32.0)

The deep-learning architectures are trained and evaluated on the test dataset. Dice scores, precision, and recall metrics are shown as an average over all classes as well as the respective class-wise values (*NE, AE, CE*). Note that with an input of two-dimensional SLA grid maps, *EddyNet* is trained on reproducing the *py-eddy-tracker* algorithm as best as possible whereas Teddy classifies eddies from unprocessed less error-prone Level 3 CMEMS along-track sea level anomaly data.

Evaluating the test dataset Teddy achieves a mean Dice score of 59.3% over all three classes, with the score outperforming that achieved from other methods such as the geometry-based algorithm *py-eddy-tracker* and the CNN-based *EddyNet* with Dice scores of 57.1% and 55.8%, respectively.

The ability to detect areas with no eddies is the highest here with a Dice score of 92.2%, which is also the case throughout all methods for this class. This performance for *NE* is also similar to other methods with a Dice score of 91.7% with the *EddyNet* and 91.3% with the *py-eddy-tracker*. The classes *AE* and *CE* are more difficult to infer correctly with Dice scores of 39.0% and 46.5%, respectively. Just as in other methods that use SLA data (Lguensat et al., 2018; Santana et al., 2020), there is a difference in the performance to detect either *CEs* or *AEs*. For our method evaluated on the region of the northern hemisphere, *CEs* are slightly easier to classify than *AEs*. However, the difference of almost 7.5% is higher than that in other research studies.

A CNN-based architecture such as the *EddyNet* performs very well with a Dice score of 87.4% when tested on a reference data set generated the same way as the training reference data set, as it was done in the study of Lguensat et al. (2018). Though it is important to note that this architecture uses the same two-dimensional SLA grid maps as an input that is used for reference data generation this architecture is rather trying to reproduce the *py-eddy-tracker* algorithm than to detect eddies more accurately. As a result, the performance of the *EddyNet* drops to 55.8% when using the test reference dataset, as there are fewer annotated eddies in the reference dataset than the highly correlated and preprocessed SLA grid maps would imply.

Comparing a sample reference and prediction such as those shown in Figures 6, it is apparent that Teddy tends to infer *AEs* and *CEs* conservatively, i.e., an eddy is often only classified when there is also a corresponding eddy in the reference data. Therefore, the recall could be a more sensitive metric to show the difference between both reference datasets. With the test reference, the model infers an output with a recall of 66.9%.

**Figure 7:**
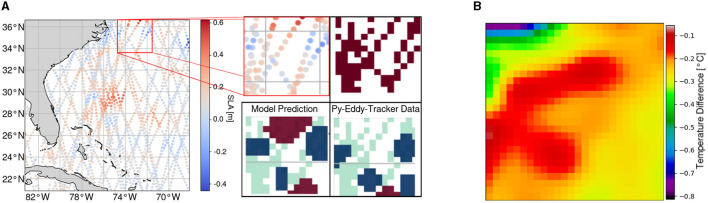
Investigation of the ability of Teddy to detect eddies, when there is only sparse along-track data available, compared to a *py-eddy-tracker* output. Teddy predicts an eddy from the input data whereas the *py-eddy-tracker* did not detect one. Sea surface temperature data independently confirm the existence of an anticyclonic eddy. **(A)** Input observations (top left), sparse feature map projection (top right), *py-eddy-tracker* output (bottom right), and Teddy prediction (bottom left) of one sample day (01.01.2017) and region with high sparsity. **(B)** Close-up of the eddy area on the sea surface temperature map, indicating an existing anticyclonic eddy.

Comparing Teddy's output sample with those from other methods shows that, in some cases, an eddy is identified by Teddy even though there was no eddy detected by the *py-eddy-tracker*. In the example shown in Figure 7, the sparsity in the area is high. The AT data show high values in this area and make it apparent by the human eye that there is indeed an eddy. However, the output from the *py-eddy-tracker* shows no annotated eddy in this area, since the SLA grid map production process made these sparse observations less apparent.

We also calculated the Dice score for only the pixels to which at least one observation of the AT data can be assigned to. The goal is to evaluate the classification performance on the AT data positions alone and how much the sparsity invariant CNN is able to infer eddies in areas where there are no AT data. Between the Dice scores evaluated on the grid map and those evaluated on the ATs, there is only a difference of 0.1%, indicating that Teddy can fill in the data gaps with semantics sufficiently. As shown in Table 1, with a mean Dice score of 46.5% and a mean recall of 44.8%, a baseline method such as the k-nn algorithm is outperformed by Teddy with a high margin since the spatiotemporal information of the input data cannot be processed. A one-dimensional baseline CNN and a state-of-the-art LSTM model infer a segmentation of SLA AT data with a Dice score of 36.8% and 49.0% and a recall of 36.8% and 41.5%, respectively. They are therefore also outperformed by Teddy only evaluated at the GT's positions (59.4%). In this section, the results of the bidirectional LSTM are presented. Despite its lower complexity, the unidirectional setup produces very similar results with a mean Dice score of 49.4% and a recall of 57.0%. The difference in performance compared to our method can again be explained by Teddy's ability to utilize the spatiotemporal information more efficiently.

**Figure 8:**
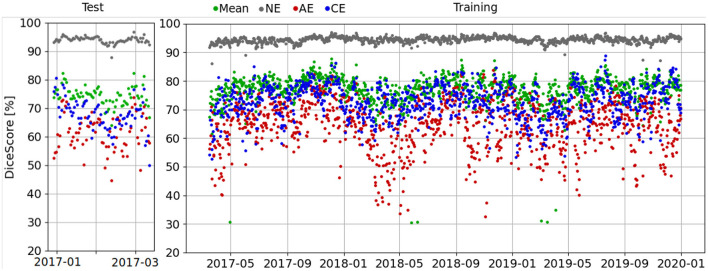
Dice scores per day evaluated in the test area for the testing time frame (**left**) and the training time frame (**right**) on the reference dataset generated for training and validation as described in Section 3.2.1. A seasonal pattern is visible in which the resulting Dice scores in spring are slightly decreased.

Furthermore, Dice scores are calculated for each day with the training reference dataset, as shown in Figure 8. Here, a seasonal pattern is visible in which the resulting Dice scores in spring are slightly decreased. We speculate that this pattern originates in the seasonally varying eddy activity.

### 5.3 Omitting the transformer module and the sparsity invariant CNN

To investigate the ability of the Transformer to process the spatiotemporal context, we infer a semantic segmentation map using only a sparsity invariant CNN architecture on the sparse feature map (see Table 2 for detailed results). Consequently, only a mean Dice score of 58.2% is achieved. The 1.1% decrease in contrast to the full Teddy architecture can be explained by the missing processing of the spatiotemporal context. For the same reason, omitting the addition of the positional encoding to the Transformer input decreases the Dice score by 1.7%. Although the spatial context is restored by the positional decoding (see Section 4.2), the time information is lost.

**Table 2:** Performances of Teddy with different modules disabled: 1. No positional encoding added, 2. Transformer module omitted, 3. Using conventional CNN instead of a sparsity invariant CNN, 4. No positional Encoding, no Transformer, and using a conventional CNN instead of a sparsity invariant CNN.

**Method**	**Dice score [*%*]**	**Precision [*%*]**	**Recall [*%*]**
w/o pos. Encoding	57.6 (91.5, 35.8, 45.4)	56.4 (93.3, 37.1, 38.7)	65.7 (90.0, 44.5, 62.7)
w/o Transformer	58.2 (91.8, 37.2, 45.7)	57.5 (93.4, 41.0, 38.0)	67.0 (90.4, 44.0, 66.5)
No spars. inv. CNN	57.6 (91.7, 36.5, 44.7)	57.0 (93.3, 39.7, 37.9)	65.9 (90.3, 44.7, 62.5)
CNN only	51.9 (90.4, 23.6, 41.8)	51.7 (92.3, 29.1, 33.5)	59.5 (88.8, 24.4, 65.4)

The metrics shown are: Mean Dice scores, mean recall, and mean precision of all classes (in brackets: *NE, AE, CE*). Every Experiment is trained newly on the training reference dataset and tested on the test reference dataset.

Due to the resulting sparsity on a projected two-dimensional grid map, we utilized the method of a sparsity invariant CNN introduced by Uhrig et al. (2017). To investigate the gain from such a network, we compared the results of an experiment with omitted sparsity invariant CNN with those from the default set-up. Here, we only reach a mean Dice score of 57.6%, showing the importance of using a CNN that specializes in sparse data.

Using a standard CNN architecture, without a Transformer module nor sparsity invariant CNNs, a Dice score of 51.9% is achieved. The temporal information is not utilized here and the spatial context is only introduced through the positional decoding. *EddyNet*, which is also a CNN-based architecture, has a similar Dice score of 55.8%. The difference here is that, while *EddyNet* has a U-Net structure, it utilizes two-dimensional SLA grid maps as an input and therefore does not have the issue of spatial data gaps.

## 6 Conclusion

In this study, we successfully demonstrated that along-track sea level anomaly data can be used to create segmentation maps of classified eddies with higher accuracy and in near real-time compared to other commonly used methods. As also mentioned, for example, in a study conducted by Santana et al. (2020), it is challenging to compare different eddy detection methods since the performance depends on the study area, data type use, characterization, and definition of eddies along with the reference data generation. Therefore, we compared our method to others by evaluating them on an independent reference dataset. In addition, we created a framework of a novel method that can rely on real-time and unprocessed satellite data, without the need of using processed CMEMS sea level anomaly grid maps that is released weeks after the measurements. This framework increases the timeliness and accuracy of our method as well as the ability to detect eddies that are hidden in geometry-based methods, such as the *py-eddy-tracker* and reduces the problem of error-prone reference data.

A promising research direction is a combination of along-track data and two-dimensional observations such as sea surface temperature or synthetic aperture radar as inputs to a multimodal network to increase accuracy. Moschos et al. (2022a) demonstrated that sea level anomaly data are not entirely reliable for classifying eddies, so it might be useful to introduce sea surface temperature maps. However, a comprehensive reference dataset that is independent and accurate is necessary for a proper evaluation. In this study, we were only able to solve this problem with a rather small test dataset. Future research will certainly help the most in evaluating the methods more accurately to detect and classify eddies. Our research area was limited to the gulf stream, whereas the behavior and patterns of eddies might differ in other regions. Another limitation is the ability to infer eddies in regions with very sparse or no AT observations. Future research could investigate Teddy's performance with varying sparsity.

## Data availability statement

The raw data supporting the conclusions of this article will be made available by the authors, without undue reservation.

## Author contributions

EB: Conceptualization, Data curation, Formal analysis, Investigation, Methodology, Software, Visualization, Writing – original draft. AA: Data curation, Investigation, Writing – review & editing. JK: Funding acquisition, Resources, Supervision, Writing – review & editing. RR: Conceptualization, Formal analysis, Funding acquisition, Methodology, Resources, Supervision, Writing – review & editing.
